# GLAD Scale for Ranking Welfare of Horses on Arrival after Transport to Slaughterhouses

**DOI:** 10.3390/ani13091465

**Published:** 2023-04-25

**Authors:** Barbara Padalino, Beatrice Benedetti, Martina Felici, Dominique Joseph Bicout

**Affiliations:** 1 Department of Agricultural and Food Sciences, University of Bologna, 40127 Bologna, Italy; beatrice.benedetti7@unibo.it (B.B.); martina.felici6@unibo.it (M.F.); 2 EPSP, Université Grenoble Alpes, CNRS, Grenoble INP, VetAgro Sup, TIMC, 38000 Grenoble, France; bicout@ill.fr

**Keywords:** journey, welfare impairment, equine, ABMs, modeling

## Abstract

**Simple Summary:**

Horses are often transported, and transport may impair their health and welfare. However, there is no tool to assess objectively their welfare after transport. This study aimed to develop the first tool for measuring horse welfare on arrival and to propose a scale to rank horses based on their welfare impairment. The tool and the scale were developed based on the literature and expert knowledge using data collected from 1019 horses traveling to three slaughterhouses. The results of a checklist containing 15 animal-based measures (ABMs), recorded on arrival, were assigned a level of welfare impairment (*S*). Based on *S*, the horses were split into four categories: good shape; light affected; affected; down (GLAD). Based on the GLAD scale, G horses can be slaughtered immediately, L horses need rest, A horses need attention and treatment, and D horses may need euthanasia or emergency slaughtering. The majority of the horses fell into the categories G (43%) and L (48%). Our tool is a simple, easy-to-use instrument to assess horse welfare after transport, specifically aimed at horses arriving at slaughterhouses, and it may assist competent authorities in deciding when a horse can be slaughtered.

**Abstract:**

To date, there is no official method for measuring horse welfare after transport. This study aimed to develop a scale to classify horses into four categories: good shape; light affected; affected; down (GLAD) based on their welfare impairment measured at unloading. To this end, 15 animal-based measures (ABMs), previously recorded from 1019 horses, were scored. Weight and severity scores provided by welfare experts, alongside the number of welfare principles highlighted by the ABM, were assigned to each ABM. The welfare impairment (*S*) of each horse was then calculated as the weighted sum of the severity scores of the 15 ABMs. Three thresholds were also set to define the four GLAD categories; the ABM “down” (i.e., horses unable to stand and walk on arrival, also considered by the law as the indicator of the worst welfare) was used as the higher threshold, *S*_down_, (category D); the intermediate threshold, *S*_2_, was defined by the ABM “injuries”, assumed to represent highly impaired welfare (category A); the threshold, *S*_1_, was defined assuming that significant welfare impairment starts from 20% of *S*_2_ (L category). Horses with an *S* value below *S*_1_ were considered physically and mentally fit (G category). Out of 1019, 43% of horses fell into category G, 48% into L, 9% into A, and 0.3% into D. Our scale could be useful for veterinarians to decide whether a horse can be slaughtered immediately (G), needs rest (L), needs attention (A), or euthanasia (D), but further validation is needed.

## 1. Introduction

Concern for animal welfare has steadily increased in recent decades [1]. Animal welfare scientists have made significant efforts towards achieving official and validated methods to assess animal welfare [2,3]. Several protocols for a proper animal welfare assessment have been developed and validated [4,5,6]. They could be used to measure welfare, develop quality certifications, identify possible risk factors, and give evidence for developing new animal welfare legislation [7]. The Welfare Quality^®^, Animal Welfare Indicator (AWIN), and the five domain protocols exist for the welfare assessment of different species [6,8,9,10,11,12,13,14,15,16]. In particular, the AWIN and the five domain approaches have been applied and validated for the equine species (*Equus caballus*) on farms [6,17].

The AWIN approach aimed to improve animal welfare conditions through the development of practical on-farm welfare assessments [6]. This tool reflected the method defined by the Welfare Quality^®^ project [5,18,19] based on four welfare principles (good feeding, good housing, good health, and appropriate behavior) and 12 distinct but complementary animal welfare criteria [5]. The protocol was composed mainly of valid, reliable, and feasible animal-based indicators, and some resource- and management-based indicators [20].

Another remarkable animal welfare approach is the so-called “5 Domains”. This was described for the first time in 1994 [4]; it is based on the Five Freedoms paradigm [21] and is designed specifically for a systematic, comprehensive, and coherent animal welfare assessment. The step forward that this approach made was to assess welfare by considering the presence of positive states instead of the absence of negative indicators [22]. This means not just identifying basic welfare needs, but also allowing further decisions to optimize animals’ physical and mental well-being [23]. With the five domains approach, welfare is no longer just avoiding negative conditions but offering animals a good quality of life through the analysis of positive indicators. Both AWIN and the five domain approaches include in their protocols mainly animal-based indicators. The importance of these indicators has been repeatedly stressed [24,25]. In fact, unlike resource- and management-based indicators, which can highlight risk factors without representing a direct welfare measure, animal-based indicators can be considered as a true welfare reflection since they monitor welfare directly from the animal [24].

The above-described protocols focus on the assessment of on-farm horse welfare. Even though transport is one of the most critical phases for horse welfare [26], there is still no official and validated method to assess the overall welfare of traveling horses [3]. One tool to assess horse welfare on arrival was proposed by Messori, and it was recently modified and applied [27,28,29]. This existing tool is based on checklists where physical, behavioral, and environmental measures are taken from the animals and the vehicles [27,28,29]. The findings of these checklists have been useful to identify several hazards for specific transport-related welfare issues, such as injuries and respiratory disorders, using statistical regression models [28,29]. Although considered as a first step to improve transport related-welfare conditions, these checklists can be time consuming and do not give an overall welfare index for the horse. In 2020, Miranda de la Lama and colleagues proposed a shorter checklist and a subsequential categorization of the horses in four clusters based on four animal-based indicators (body condition score, coat quality, prevalence of lameness, and ocular and nasal discharge) assessed on arrival at two slaughterhouses in Mexico [30]. However, the study does not report any calculation to assess the overall welfare index of the horses [30].

Considering the knowledge gaps described above, this study aimed to develop a tool to determine an overall welfare score for horses at unloading and to create a scale (GLAD scale) for the categorization of the horses into different welfare states. As an illustrative application and working example of the approach, data collected from over a thousand horses transported to slaughterhouses were used.

## 2. Materials and Methods

### 2.1. Data Collection

Data collection was performed from May 2021 to May 2022 for horses traveling on 52 commercial journeys. A total of 1019 horses transported from different countries to three different Italian slaughterhouses, 2 in southern and 1 in northern Italy, were used. On arrival at slaughterhouses, the welfare status of the horses was assessed following the protocol described by Zappaterra et al. [29] (modified from Messori et al. [27]). Briefly, the assessment was performed 3 times, filling in 3 specific checklists during unloading, 30 min after, and 24 h after unloading. The checklists included animal-based measures (ABMs) and environmental-based measures (EBMs) recorded at 3 levels: driver, vehicle, and animal.

### 2.2. Data Handling

The research group organized several meetings to discuss the ABMs to be included in the GLAD scale. Among the ABMs previously collected, the authors selected ABMs based on the recent literature used in establishing the European Food Safety Authority (EFSA) scientific opinion [3]. Expert judgement of the suitability of these ABMs assessed the impaired, or not, welfare status of the horse on arrival. In the end, a total of 15 ABMs were selected and each ABM was scored (Table 1).

For an animal “*n*” the score $a_{n,m}$ in relation to ABM “*m*” is defined as indicating both presence/absence of the ABM under consideration and its severity (low/moderate/high). In this study, we used the following 3-level scale as follows (Table 1): $a_{n,m}$ = 0, 1, and 2, for absent, present–moderate, and present–high, respectively. Some of the scores initially recorded as present/absent (1/0) were rescored (i.e., rs.scores) based on their severity to give an equivalent weight (Table 2).

### 2.3. Formulas

This section details the formulas used for calculating the impairment score and the rationale for setting the thresholds of the GLAD scale.

#### 2.3.1. ABM Weight, $w_{m}$

**Figure 1:**
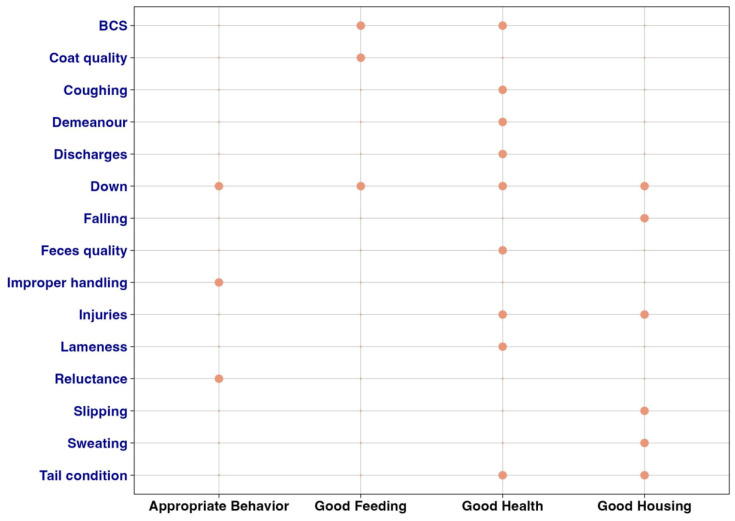
Number of welfare principles impaired by the presence of the ABM. Relational association between ABMs and welfare principles (good behavior, good feeding, good health, good housing) suggested by expert knowledge. Orange dots should be interpreted as the association between the ABM and the welfare principle impairment. The sum of orange dots for each ABM represents $k_{m}$.

**Figure 2:**
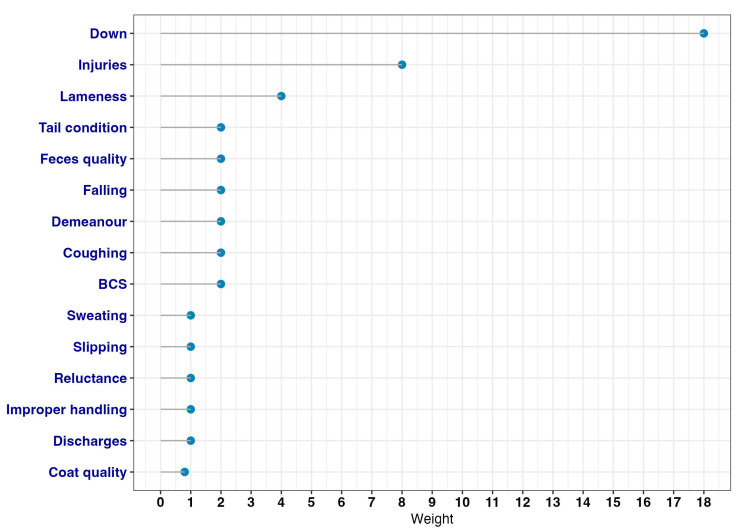
ABM weights calculated by multiplying *b_m_* × *k_m_* as described in Section 2.3.1.

After choosing the 15 ABMs to consider, a weight was given to each of them. The weight $w_{m}$ of an ABM *m* gives the relative magnitude (compared to other ABMs of the same checklist) of the contribution of this ABM to the total score of welfare impairment. Thus, $w_{m}$ also allows ranking the ABMs. The $w_{m}$ has been constructed as follows:

$$w_{m}=b_{m}\times f\left(k_{m}\right)$$

where $b_{m}$ is the assigned intrinsic weight (assigned by the knowledge of 3 experts (BP, MF, DJB) with consensus) (Table 2) and $f\left(k_{m}\right)$ (with $f\left(k_{m}=1\right)=1$) a function of $k_{m}\;$, which is the number of welfare principles (good behavior, good feeding, good health, good housing) impaired when the ABM was present. The number of impairments was suggested by expert knowledge (Figure 1). For simplicity, the linear function, $f\left(k_{m}\right)=k_{m}$ was used. In addition, feasibility, sensitivity, and specificity of the ABM were considered by the experts when suggesting the $b_{m}$. The ABM weights are graphically shown in Figure 2.

#### 2.3.2. Welfare Impairment Score, *S*

To give a final score for each animal, the following formula was used where N was the number of each animal to be checked in relation to the list of M ABMs. The welfare impairment score $\;S_{n}$ for any animal *n* (with, *n* = 1,⋯,N) is given by:

$$S_{n}=\overset{M}{\underset{m=1}{\sum }}a_{n,m}w_{m}$$

where $a_{n,m}$ is the ABM score (see Table 1) of animal “*n*” and $w_{m}\;$ is the ABM weight (see Section 2.3.1.). As defined, the set of $a_{n,m}\;$ represents the ABM spectrum of an animal and $S_{n}\;$ is the weighted cumulative sum of ABM scores. The lowest and highest scores are $\;S_{n}\;$= 0 (absence of welfare impairment) and $S_{n}\;$= M×”max” ($a_{n,m}$ ), respectively.

#### 2.3.3. GLAD Scale Construction

To construct the GLAD scale, 4 characteristic states were considered:

- **Good shape:** the animals are not at all or are affected very little after transport, so they can be taken directly to the slaughterhouse without any actions.
- **Lightly affected:** the animals have slight welfare disorders for which a restorative rest in the lairage would suffice before slaughtering.
- **Affected:** the animals are sufficiently affected after transport that their welfare state requires treatment actions to restore it to better conditions for the slaughterhouse.
- **Down:** the animals are seriously affected after transport. The horses cannot stand and walk on arrival, they are exhausted. Competent authorities must treat them immediately. The animal must be stunned immediately in the vehicle and then be transferred to the slaughterhouse mechanically to complete the procedure. The meat has to be checked carefully [34].

A welfare impairment degree (WID), *d*, has been developed to grade the degree of impairment. For an animal, the WID can be defined as the measure of the extent to which the welfare has been impaired as:

$$d=100\;\left(\frac{S}{S_{down}}\right)$$

where *S* is the welfare impairment score of the horse (see Section 2.3.2) and $S_{down\;}$ is the score for a horse in the down state (the worst status). By definition, *S* varies between 0, corresponding to the absence of impairment, up to a maximum value. As *S* ≥$\;\;S_{down}$ corresponds to animals in the down state (D), 2 threshold scores, $S_{1}$ and $S_{2}$ (with $S_{1\;}$ < $\;S_{2}\;$ < $\;S_{down}$), were defined for classifying animals in G, L, and A states (Table 3), respectively, using the following parameters:

$$r_{1}=S_{1}/S_{down}\;\mathrm{and}\;r_{2}=S_{2}/S_{down}$$

#### 2.3.4. Thresholds $S_{1}$, $S_{2}\text{, }$ and $S_{\mathrm{down}}$

To estimate the welfare impairment degree there was the need to decide the values of the thresholds which were discussed and agreed upon during research team meetings. The thresholds were defined as follows:

- $S_{down}$: Based on an inventory of ABMs mostly found on animals in the down state (as defined above), $S_{down}\;$can be considered as the (nonzero) minimum of welfare impairment scores of animals in that situation.

- $S_{2}$: As $S_{down}\;$ is the lower band of the worst-case status, and $S_{2}\;$ divides the range, 0 < *S* < $\;S_{down}$, into 2 parts: that of scores *S* < $\;S_{2}$ corresponding to states with less-alarming welfare impairments, and that of scores *S* ≥ $\;S_{2}$ representing welfare so altered that veterinary actions are required. Therefore, $S_{2\;}$ can be set as the (nonzero) minimum of welfare impairment scores for target ABMs.

- $S_{1}$: Finally, $S_{1\;}$ can be defined as a proportion of *S*_2_ below which the impairment status of welfare can be considered acceptable for slaughter.

The quantitative determination of thresholds was then undertaken as follows:

- $S_{down}$: the ABM “Down” was used as representative of the down state. Indeed, animals with downing troubles are in such bad shape that they tick all the boxes of welfare concerns, affecting all 4 welfare principles. The welfare impairment score corresponding to a downer (i.e., a horse with the ABM down) is: $a_{down}\times w_{down}=2\times 18=36$. Furthermore, animals below (with no apparent down state) but close (with several other ABMs) to this threshold can be included in the down state for veterinary officer (VO) intervention. To this end, we used:

$$S_{down}=80\%\times a_{n,down}\times w_{down}=0.80\times 36=28.8\approx 29$$

In summary, the down class (D) consists of animals with downer and non-downer animals with several severe ABMs that require a VOs intervention.

- $S_{2}$: The ABM “Injuries” was assumed to represent highly impaired welfare requiring immediate actions or interventions. Then, *S*_2_ was chosen at 90% of the highest score associated with injuries as:

$$S_{2}=90\%\max\left(a_{injuries}\right)\times w_{injuries}=0.9\times 2\times 8=14.4\approx 14$$

- $S_{1\;}$: It was assumed that significant impairments start from approximately 20% of $S_{2}$, i.e.:

$$S_{1}=20\%\;S_{2}=0.20\times 14=2.8\approx 3$$

Table 4 explains the categories used for the GLAD scale based on the calculated thresholds. Categories were defined considering the formulas reported in Table 3.

## 3. Results

### 3.1. Horse Welfare Impairment Degree (WIDs)

Out of 1019 horses, upon arrival the majority of the horses fell into category G (Table 5). Moreover, among the 43% of horses in category G, 45/1019 (4%) did not have any welfare impairment, that is, all ABMs were scored “zero” (i.e., *d* = 0).

**Figure 3:**
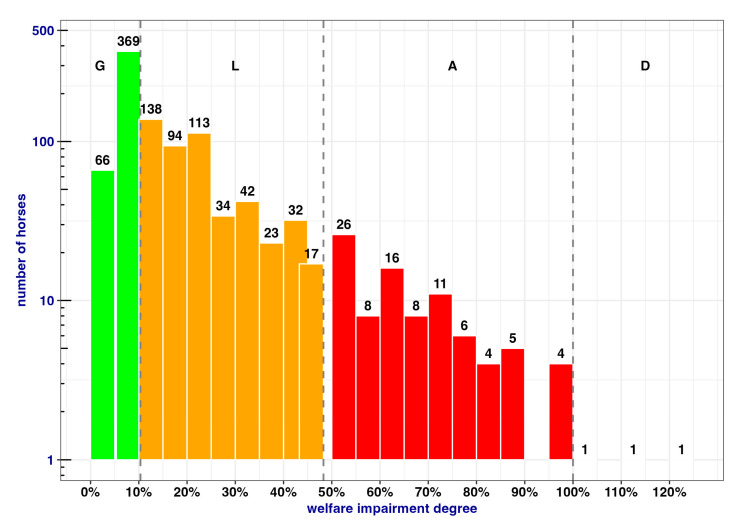
Overall distribution of horse WIDs in the 1019 horses considered. Dashed vertical lines indicate threshold values. The three horses in the down state are indicated by the quoted numbers.

The overall distribution and color-coded differentiation according to the GLAD scale scores are shown in Figure 3.

**Figure 4:**
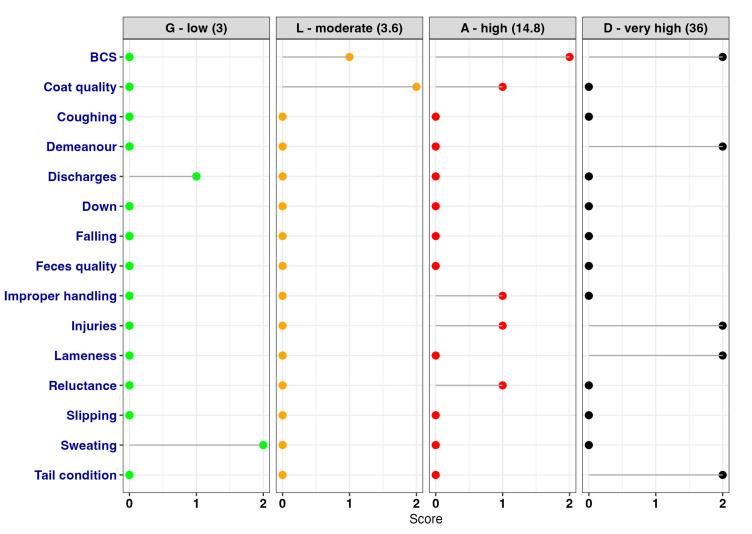
Examples of different spectra for each GLAD category. Numbers between parentheses correspond to welfare impairment final scores (*S*). Final scores were given as reported above, considering the ABMs assessed and their weight (see Section 2.3.2).

Considering that 974 horses (1019–45, where 45 horses had score 0) have been scored with “nonzero”, the number of spectra is quite large. Figure 4 illustrates an example of spectra per each GLAD category.

### 3.2. Distribution and Prevalence of Animal-Based-Measurements (ABMs)

**Figure 5:**
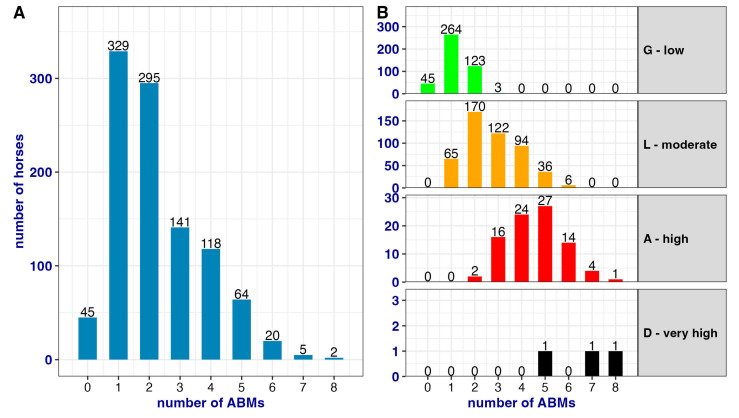
Distribution of the number of ABMs assessed on horses. Panel (**A**) indicates the general frequency of the number of ABMs assessed, regardless the category. Panel (**B**) indicates how the number of ABMs is distributed in the four different GLAD categories.

The general and per GLAD category distribution of ABMs is reported in Figure 5. In the G horses, a maximum of three ABMs were recorded, and the most frequent ABMs reported were BCS, coat quality, slipping, and discharges. In the L horses, up to six ABMs were recorded, and the most frequent were BCS, coat quality, tail condition, improper handling, and reluctance to move/unload. In the A horses, on average five ABMs were recorded, but four horses showed seven ABMs and one horse showed eight ABMs. The most common ABMs scored in this category were injuries, BCS, coat quality, and tail condition. Finally, in the D horses, a minimum of five and a maximum of eight ABMs were found, and the most common ABMs for this category were BCS, tail condition, injuries, and demeanor. The total number of ABMs recorded for each horse may indicate the welfare impairment and the possible category in which the horse falls after the calculation.

**Figure 6:**
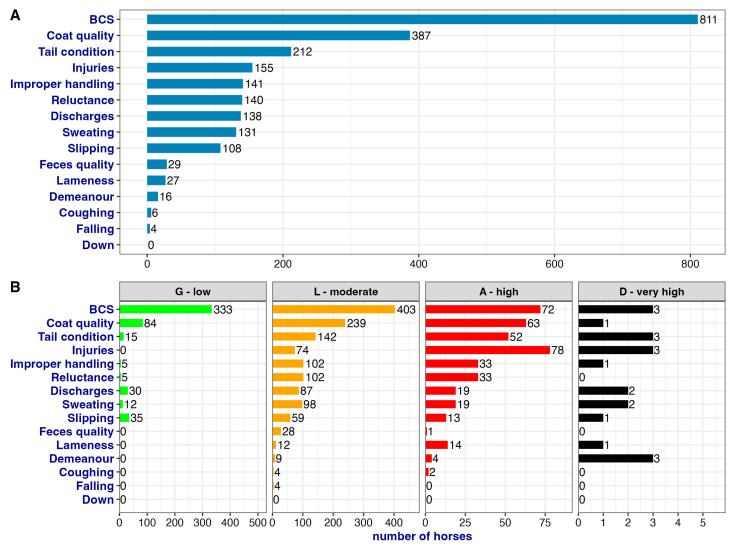
Prevalence of single ABMs. Panel (**A**) hows the overall prevalence of each ABM, i.e., how many horses were scored with the indicated ABM. Panel (**B**) shows the prevalence within each GLAD category.

The general prevalence of each ABM and its prevalence within each category of the GLAD scale are reported in Figure 6. It is important to notice that BCS was one of the most recorded ABMs, found in 80% of cases (811/1019), and it was well distributed in all four categories. Similarly, improper handling was present in all categories but had a lower total prevalence (13.8%). While the ABM falling was detected in four horses that were all included in the L category, the assessment of ABM lameness increased with the severity of the category, and overall, it was reported in 2.7% of cases (27/1019). The ABM injuries had a total prevalence of 15% (155/1019) and it was more represented in the A and D categories. Finally, even though there were no horses that arrived non-ambulatory or exhausted (i.e., with the ABM down), three horses fell into the D category. This was due to the added effects of other ABMs resulting in the equivalent of a downed horse.

## 4. Discussion

This study aimed to develop a tool to objectively assess the overall welfare of horses after road transport to slaughterhouses and to propose the GLAD scale to decide when and if to slaughter the traveling horses. Based on the total welfare impairment score calculated, it was possible to place horses into four distinct categories (G, L, A, D). These categories are crucial to objectively define the future of the animal; therefore, our scale could be useful for competent authorities to decide whether a horse can be slaughtered immediately or needs rest, veterinary care, or emergency stunning/euthanasia.

To construct the scale, 15 different ABMs were selected. The decision to include exclusively ABMs and no other indicators was dictated by EFSA’s recent explanations of the importance of these measures in animal welfare assessment [24]. It is stated that ABMs are the most appropriate indicators of animal welfare and a careful selection and combination of them can be used to assess the welfare of a target population in a valid and robust way [24]. Since the most appropriate combination of ABMs will depend on the purpose of the welfare assessment [24], in our case, the selection considered ABMs which have been previously tested in other horse welfare protocols [20,27] and were chosen by a group of experienced researchers based on the most recent evaluation of transport-related literature [3]. ABMs were screened and retained in the final model to create an effective, fast, and easy-to-use tool.

ABMs should be measurable, valid, and feasible [24]. ABMs that were previously reported as crucial in measuring horse welfare, such as BCS and coat quality, were therefore included in our tool, as they were previously judged feasible and valid indicators [20,35]. In 2020, Miranda de la Lama et al. [30] also considered BCS and coat quality to assess a horse’s fitness for transport and interestingly found that they were useful to characterize the fittest horses but were less effective in profiling a worse welfare status. On the contrary, ABMs including lameness and nasal or ocular discharge revealed a greater sensitivity to discriminate the worst welfare profiles and were considered as appropriate “iceberg indicators” [30]. For this reason, in our study all the above-mentioned ABMs were included, but to develop the ‘overall welfare score’ a different and objective weight was attributed to each of them as they may affect the welfare status differently. In particular, their weight as a representation of the severity of welfare impairment was attributed by experts considering the number of welfare principles impaired by the presence of that ABM.

One of the study’s main results was to structure a clear scale based on the welfare impairment caused by transport. Defining thresholds a priori can be challenging. In this study, the choice of thresholds has been based on a good knowledge of the relationships between ABMs and welfare. To the authors’ knowledge, no study has previously provided threshold scores to differentiate the general condition of horses after transport. In 2020, Miranda de la Lama and colleagues [30] aimed to identify different clusters of horses considering their fitness on arrival at a slaughterhouse. Clusters were constructed with a two-step cluster analysis and the final analysis was made using only four ABMs. These clusters represented only the analyzed population and cannot be applied to other populations and scenarios. On the contrary, in the present study, the category thresholds were calculated with a mathematical model and represented a universal way to assess horse welfare after any transport, independent from the reason for or the length of the journey.

In our study, one of the thresholds taken as a reference was the ABM injuries. The presence of injuries has been considered one of the most important ABMs in identifying welfare impairment of animals [25]. Consequently, in our study, the prevalence of injuries increased as the welfare impairment within the four categories (G, L, A, D) increases. It resulted in more injuries in categories A and D. This confirms what was reported in other studies [20,25], namely that this ABM is one of the most important in identifying the welfare status of the horses. In our study, on average 15% of horses reported at least one injury. This is lower than the prevalence reported by Marlin et al. [36], where 24% of horses transported in Italy reported at least one recent injury but is in line with what was registered by Roy et al. [37] in Iceland. To maintain a low prevalence of injuries at unloading it is important to assess the horse’s fitness for transport, as explained by the European guidelines [38]. In fact, as recently explained by the EFSA report on equidae transport, the pain associated with any disease before the journey will affect the horse’s emotional state and will predispose it to further injuries or pathologies during the journey [3].

In conjunction with the low prevalence of injuries we found, the ABM improper handling was only reported in a few cases. In fact, other studies found that horses subjected to punishment by handlers had an increased number of injuries and poor welfare outcomes [39]. For this reason, the ABM improper handling was included in the GLAD scale because it is considered one of the most important transport-related risk factors for its consequences on horse welfare [3]. To prevent welfare impairment caused by improper handling, self-loading and self-unloading training for horses is highly recommended to reduce fear and stress associated with these procedures [40]. Moreover, it reduces the occurrence of human–horse interactions [40] and the consequent risk of human–horse interaction injuries [41].

In the GLAD scale, the ABM down was considered as the maximum welfare impairment and the *S*_down_ threshold was set using this ABM. Nevertheless, in this study, there were no horses found down or exhausted on arrival. This indicates that downer horses, fortunately, are not so common in the European slaughterhouse trade. In another study, the incidence of downer horses was detected, and the authors suggested that the presence of these animals was due to important welfare issues with either the fitness for transport and/or the journey conditions [42]. Our considerations were also made by comparing horses with other species, i.e., cows, where downer animals are frequent and indicate very poor welfare conditions at unloading [43]. In fact, in this situation, the law demands emergency slaughter directly in the vehicle to prevent the animal from suffering [34]. In horses, the downer condition could occur when the load is crowded (i.e., a horse falls and is trampled) or could also be due to the “exhausted horse syndrome” that occurs when a horse has undertaken prolonged exercise and experiences fatigue, hyperthermia, and water and electrolyte loss [32,44], which are all conditions that can occur during or after a long journey. Roy et al. [42] found that horses were more likely to develop this condition during long travel periods (32 h) from the USA to Canada. In our study, the horses came from various parts of Europe and had travelled for lengthy periods but none of them arrived in this condition. This difference is probably because, apart from journey durations, the horses in this study traveled in compliance with the requirements of the EC 1/2005 [45], which regulates that horses must be watered and fed every 8 h and unloaded after a maximum journey duration of 24 h. The journey conditions enforced by this regulation seem to reduce the risk of downer animals.

By applying the GLAD scale to the dataset of this study, it was found that the majority of the horses fell into the G and L categories. These results indicate that the majority of horses were in a generally good state, did not need any veterinary attention, and could be slaughtered immediately or shortly after arrival. This supports previous studies undertaken using these types of datasets with horses [28,29], namely that they arrived in overall good health and welfare conditions. Nonetheless, our results are in contrast with what was reported by Marlin et al. [36], where 37% of horses that arrived at the slaughterhouse after a long-distance journey were deemed unfit for transport. The horses in our study travelled for long journeys too, but, despite this, arrived with a good welfare status. This suggests that the assessment of fitness for transport and journey conditions, and consequently the welfare status of horses transported across Europe, have improved in the decade after the enforcement of the EC 1/2005 [45]. However, it is also worth highlighting that our findings may be underestimated as the drivers and slaughterhouse owners agreed to this study, so our presence may have minimized rough and improper handling during unloading.

Although most of the horses in our study had a low GLAD score, horses with higher scores have been recorded. In the latter horses, which tend to fall into the A or D categories, we mostly assessed a high number of ABMs, with five or more. In fact, independent from the weight given to each ABM, the more ABMs there are, the more the horse’s welfare is impaired. This could help official veterinarians (OVs) to complete an initial screening of the horses before scoring them definitively. Indeed, it is possible to state that horses with ≥5 ABMs need special consideration and further assessment while horses with <5 ABMs are likely to have a less alarming welfare status. However, it is worth considering that counting only the number of ABMs is not a good evaluation of welfare impairment as all ABMs do not reflect the same level of impairment but can be used only as an initial filter. Welfare impairment must be properly calculated as described and each horse categorized in the GLAD scale. Further studies aiming to create an automatic calculation of the welfare impairment (e.g., GLAD app) may be of interest to simplify the work of the welfare officer on arrival.

In our study, the GLAD scale was used to assess horses that were destined for slaughter. The EU legislation 1099/2099 [34] defines that if animal welfare and/or health is somehow impaired, the horse should rest and receive veterinary care before being slaughtered. Furthermore, animals that are not able to stand at unloading should be euthanized or emergency slaughtered, and their meat should be analyzed in a post-mortem exam to get permission to be sold [34]. The boundaries within which these laws are applied are difficult to define, mainly because OVs have no valid tools that objectively define the status of the animal to help them make the decision. The GLAD scale could become this tool.

This study defines, for the first time, a functional tool for the assessment of horse welfare at unloading. Nevertheless, it still has some limitations. First, the calculation of this study is based on a dataset where the ABMs were scored by a trained and expert assessor, but untrained assessors may misjudge the ABMs, and, consequently, the welfare impairment of the horses. A further study will be conducted in which several sets of horses will be evaluated by different assessors to capture both the inter- and intra-variability and repeatability of the ABM scoring. Second, in a top-down approach, we used a priori thresholds to classify the horses in GLAD. In a bottom-up approach, the work would consist of using empirical data on the dispatching of horses on arrival at the slaughterhouse to build the thresholds and outline a validation. Third, the entire study is based on a population of horses exclusively destined for Italian slaughterhouses. Most of these horses were draft horses, so different breeds are not equally represented in the sample. In follow-up studies, the authors aim to overcome these limitations by including numerous breeds and considering several types of travel (e.g., towards competitions), different journey conditions (i.e., space allowance and the ability of the horse to balance in transit), and by validating the scale with a gold standard (e.g., expert judge, hormonal analysis, etc.). With these future improvements it will be possible to validate the GLAD scale. The future aim will be to strengthen this tool and make it usable for the objective measurement of horse welfare at unloading, possibly also using technological support (i.e., GLAD app). However, considering that transportation is one of the most stressful events in an animal’s life, it represents a major welfare concern [46]. Horses are one of the most versatile species [20] and are among the most transported animals in Europe [47]. For different purposes and destinations, further validation of the scale in different transport-related conditions is needed. This could make the GLAD scale a tool to objectively assess the welfare at unloading of all horses and to make quick but valid decisions on the horse’s future after any journey.

## 5. Conclusions

This study developed, for the first time, a tool to assess the welfare of horses at unloading. It groups them into four distinct categories that indicate their possible needs depending on their overall welfare impairment. The construction of the scale, which includes 15 selected ABMs, and the definition of the thresholds were the main results of the study. The GLAD scale, applied to 1019 horses upon arrival at slaughterhouses, was revealed to be an effective and easy-to-use tool. The placement of the horses among the four categories accurately reflected the horse transport scenario in Europe, confirming good general journey conditions and welfare status on arrival. No downer horse was detected and only three horses fell into category D. In conclusion, the GLAD scale is a useful tool for the overall welfare assessment of horses at unloading and it can help OVs to screen the animals and decide, according to their welfare impairment, what to do with each of them. However, further studies are necessary to validate the scale and to test it on different and larger horse populations. The ultimate aim will be to make this tool usable at unloading regardless of a horse’s destination.

## Figures and Tables

**Table 1:** Name, description, and score explanation of the 15 ABMs included in the GLAD scale.

ABM	Description	Score Explanation
Body condition score (BCS)	The amount of stored fat in horse’s body. Scored from 0 (very poor) to 5 (obese) [31].	0 = BCS of 3 1 = BCS of 2 or 4 2 = BCS of 0, 1, or 5
Coat quality	Coat conditions (i.e., opaque, dry, dirty, clean, shiny) [6].	0 = clean/shiny coat 1 = slightly dirty/dry coat 2 = dirty/opaque coat
Coughing	A sudden and noisy expulsion of air from the lungs [27].	0 = absence of coughing 1 = presence of coughing
Demeanour	State of the sensorium and reactivity to external stimuli (i.e., alert/responsive, alert/quiet, lethargic, depressed, exhausted, scared) [29].	0 = positive demeanor (alert, responsive or alert, quiet) 1 = negative demeanor (lethargic, depressed, exhausted, scared)
Discharges	Loss of material from the nose or other orifices (i.e., eyes, penis, vagina, etc.) [6].	0 = absence of discharges 1 = presence of unilateral nasal discharge or lacrimal discharge or genital discharge 2 = presence of bilateral nasal/lacrimal discharge or combined nasal and other discharges
Down	The horse cannot rise or is unable to stand or walk unaided [27], “exhausted horse syndrome” [32].	0 = normal ambulation 1 = no ambulation
Falling	A loss of balance during unloading causing any part of the body (other than hooves) to touch the ground [27].	0 = no falling during unloading 1 = at least 1 falling during unloading
Feces quality	Feces consistency (i.e., watery, diarrhea, normal, compact and dry) [6].	0 = normal feces 1 = abnormal feces (watery, compact and dry) or diarrhea
Improper handling	Hit with the hand with a moderate to high use of force (i.e., a distinct sound heard) or hit with a stick with moderate to high use of force [33].	0 = horse being unloaded without being hit with hand or stick 1 = horse being hit with hands 1, 2, or 3 times 2 = horse being hit more than 3 times with hands and/or being beaten with a stick
Injuries	Cut(s) or wound(s) more or less severe on the horse’s body [29].	0 = absence of cuts/injuries 1 = presence of 1 cut/injury 2 = presence of 2 or more cuts/injuries
Lameness	A slight or modest attempt to take the weight off one or more limbs [29].	0 = absence of lameness 1 = presence of lameness
Reluctance to move/unload	Unwillingness to go forward (not caused by physical obstructions) or suddenly stopping just before the beginning or during unloading for at least 3 s [27].	0 = absence of reluctance to move/unload 1 = presence of reluctance to move/unload
Slipping	A loss of balance during unloading, without any part of the body (other than hooves) touching the ground (i.e., falling) [27].	0 = no slipping during unloading 1 = at least 1 slip during unloading
Sweating	Wet coat, dried sweat spots, or salt deposits [27].	0 = no sign of sweating 1 = sweating signs on part of the body 2 = sweating signs on the entire body
Tail condition	Presence or absence of ruffled/injured tail [29].	0 = no damaged tail 1 = ruffled tail 2 = open skin lesions on the tail

**Table 2:** Scores and weights used for each ABM in this study. Scores are from Table 1, rs.scores are rescaled scores (if any), *b_m_* is assigned intrinsic weights and *k_m_* from Figure 1, where *b_m_* and *k_m_* are defined in Section 2.3.1. The intrinsic weights were discussed by 3 experts who agreed on these values.

ABM	Scores	rs.Scores	*b_m_*	*k_m_*	$\mathrm{Weight}\;(w_{m})$
BCS	0, 1, 2	0, 1, 2	1.0	2	2.0
Coat quality	0, 1, 2	0, 1, 2	0.8	1	0.8
Coughing	0, 1	0, 2	2.0	1	2.0
Demeanour	0, 1	0, 2	2.0	1	2.0
Discharges	0, 1, 2	0, 1, 2	1.0	1	1.0
Down	0, 1	0, 2	4.5	4	18.0
Falling	0, 1	0, 1	2.0	1	2.0
Feces quality	0, 1	0, 2	2.0	1	2.0
Improper handling	0, 1, 2	0, 1, 2	1.0	1	1.0
Injuries	0, 1, 2	0, 1, 2	4.0	2	8.0
Lameness	0, 1	0, 2	4.0	1	4.0
Reluctance to move/unload	0, 1	0, 1	1.0	1	1.0
Slipping	0, 1	0, 1	1.0	1	1.0
Sweating	0, 1, 2	0, 1, 2	1.0	1	1.0
Tail condition	0, 1, 2	0, 1, 2	1.0	2	2.0

**Table 3:** Categories of the GLAD scale calculated based on the estimated welfare impairment degree.

WID (d)	Welfare Impairment	Color Code	State
$0\leq d<100\;r_{1}\;\%$	low	green	**G**ood shape
$100\;r_{1}\%\leq d<100\;r_{2}\%$	moderate	orange	**L**ightly affected
$100\;r_{2}\%\leq d<100\;\%$	high	red	**A**ffected
d≥100%	very high	black	**D**own

**Table 4:** Categories of the GLAD scale proposed in this study, based on the welfare impairment degree, including their color code and meaning (i.e., state).

WID (d)	Welfare Impairment	Color Code	State
0≤d<10 %	low	green	**G**ood shape
10%≤d< 48%	moderate	orange	**L**ightly affected
48%≤d<100%	high	red	**A**ffected
d≥100%	very high	black	**D**own

**Table 5:** Number (proportion) of horses in percentage assessed on arrival at three Italian slaughterhouses applying the GLAD scale.

WID (d)	N of Horses (%)	Welfare Impairment	State
0≤d< 10%	435 (43%)	low	**G**ood shape
10%≤d< 48%	493 (48%)	moderate	**L**ightly affected
48%≤d<100%	88 (9%)	high	**A**ffected
d≥100%	3 (0.3%)	very high	**D**own

## Data Availability

The data presented in this study are available upon request from the corresponding author.
